# Associations of cerebrospinal fluid profiles with severity and mortality risk of amyotrophic lateral sclerosis

**DOI:** 10.3389/fnins.2024.1375892

**Published:** 2024-05-15

**Authors:** Jiajia Fu, Xiaohui Lai, Qianqian Wei, Xueping Chen, Huifang Shang

**Affiliations:** ^1^Department of Neurology, West China Hospital, Sichuan University, Chengdu, China; ^2^Rare Disease Center, West China Hospital, Sichuan University, Chengdu, China; ^3^Laboratory of Neurodegenerative Disorders, West China Hospital, Sichuan University, Chengdu, China

**Keywords:** amyotrophic lateral sclerosis, cerebrospinal fluid profiles, disease phenotype, IgG, Q_ALB_ (CSF albumin/serum albumin)

## Abstract

**Background:**

The relationship between routine cerebrospinal fluid (CSF) testing and the disease phenotype of amyotrophic lateral sclerosis (ALS) is unclear, and there are some contradictions in current studies.

**Methods:**

This study aimed to analyze the relationship between CSF profiles and disease phenotype in ALS patients. We collected 870 ALS patients and 96 control subjects admitted to West China Hospital of Sichuan University. CSF microprotein, albumin, IgG, index of IgG (IgG_index_), albumin quotient (Q_ALB_), and serum IgG were examined.

**Results:**

In ALS patients, CSF IgG, and Q_ALB_ were significantly increased, while CSF IgG_index_ was decreased, compared with control subjects. Approximately one-third of ALS patients had higher CSF IgG levels. The multiple linear regression analysis identified that CSF IgG_index_ was weakly negatively associated with ALS functional rating scale revised (ALSFRS-R) scores (*β* = −0.062, *p* = 0.041). This significance was found in male ALS but not in female ALS. The Cox survival analyses found that upregulated CSF IgG was significantly associated with the increased mortality risk in ALS [HR = 1.219 (1.010–1.470), *p* = 0.039].

**Conclusion:**

In the current study, the higher CFS IgG was associated with increased mortality risk of ALS. CSF IgG_index_ may be associated with the severity of ALS. These findings may be sex-specific.

## Introduction

Amyotrophic lateral sclerosis (ALS) is a neurodegenerative disease characterized by loss of upper motor neurons and lower motor neurons resulting in progressive muscle atrophy and paralysis (Yang et al., 2022; Akçimen et al., 2023). The ALS phenotype varies across different ethnicities and regions. The average age of onset in ALS cohorts is around 66 years in Germany, 52 years in northern China, and 55 years in southwestern China (Rosenbohm et al., 2018; Chen et al., 2021; Wei et al., 2022). Currently, the majority of research suggests that ALS is associated with various factors such as immunity and infection, metallic elements, genetics, environmental factors, and many others (Li C. et al., 2022; Yang et al., 2022, 2023). However, the etiology and pathogenesis of ALS are highly complex and remain unknown. There are regional and racial differences in ALS patients. The research of biomarkers in cerebrospinal fluid (CSF) is one of the most active areas of ALS. Most previous studies focused on comparing the differences between healthy controls (HCs) and ALS patients, and most found that CSF IgG levels, CSF protein levels, and the quotient of CSF albumin and blood albumin (Q_ALB_) in ALS patients were higher than those in controls (Gårde et al., 1971; Leonardi et al., 1984; Norris et al., 1993; Assialioui et al., 2022). However, the associations between CSF profiles and the disease phenotype of ALS are controversial. Some studies found that the CSF protein of patients with late-onset ALS was significantly higher than that of patients with early-onset ALS (Guiloff et al., 1979; Leonardi et al., 1984), while other studies found that the CSF protein of ALS patients gradually decreased with the increased age of onset (Guiloff et al., 1980; Norris et al., 1993). Some studies found that ALS patients with higher CSF IgG, CSF protein, and Q_ALB_ had short survival times (Guiloff et al., 1980; Li J. Y. et al., 2022; Klose et al., 2023). However, some studies did not find that CSF protein levels were associated with the survival of ALS (Norris et al., 1993; Li J. Y. et al., 2022). Besides, the associations with the disease phenotypes such as the stage of the disease or the progression rate turned out to be unapparent in the majority of the studies (Tarasiuk et al., 2012; Zhao et al., 2020). One recent study found CSF protein was significantly negatively associated with the ALS functional rating scale revised (ALSFRS-R) scores in female ALS, not in male ALS (Assialioui et al., 2022). These inconsistent findings of the CSF profiles may result from inherent limitations, including small sample size, varied applied methods, characteristics of enrolled ALS patients, ethnic differences of participants, and so on. Therefore, the relationship between CSF profiles and disease phenotypes needs further study.

As the basic laboratory examinations, CSF profiles might reflect the pathophysiological alterations along the disease course and provide insight into the pathogenesis of the disease. Therefore, in the present study, we analyzed the CSF profiles from a longitudinal cohort of ALS from Southwest China, trying to elucidate the relationship between the CSF profiles and the phenotype, severity, progression, and prognosis of the disease.

## Methods

### Patients and controls

#### Inclusion criteria

All ALS patients included in the study, from the Department of Neurology, West China Hospital of Sichuan University between 2009 and 2022, who received a diagnosis of probable or definite ALS based on the El Escorial revised criteria. Demographic and clinical characteristics, such as age of onset, sex, disease type, disease stage, site of onset, disease course, progression rate, ALSFRS-R scores, and survival status, were recorded for each patient. Control subjects were admitted to the hospital for suspected neurological diseases, and underwent lumbar puncture as part of routine diagnostic procedures; no medication was administered at the time of the lumbar puncture.

#### Exclusion criteria

In the present study, participants with acute infections or trauma were excluded. Furthermore, individuals with immune system-related conditions such as multiple sclerosis, systemic lupus erythematosus, etc., which could potentially impact immune-related factors, were also excluded. ALS patients who did not undergo complete follow-up were excluded from the primary analysis; their data was solely utilized for sensitivity analysis.

The disease course was defined as the interval between the first symptoms of the disease onset and the hospitalization when the CSF and serum were examined. The progression rate was calculated using the total revised ALSFRS-R and symptom duration at diagnosis. ALSFRS-R scores were spliced into three subgroups of severity: mild (37–48), moderate (25–36), and severe (0–24). All the ALS patients were followed up at about 3-month intervals, and it was possible to determine a slope of deterioration for the clinical features of these patients. For survival analysis, the database was closed in August 2023. The term “death” is defined as death, endotracheal intubation, tracheostomy, and date of death confirmed by relatives.

We received approval from the ethical standards committee on human experimentation at West China Hospital. Written informed consent for research was obtained from all patients and control subjects participating in the study. All procedures and protocols were carried out in accordance with the guidelines of the Declaration of Helsinki and the International Ethical Guidelines for Human Biomedical Research.

#### CSF analysis

Lumbar puncture was performed in all participants. All CSF samples were checked for blood contamination. No sample was excluded due to contamination. All tests were conducted by the Department of Experimental Medicine of West China Hospital. Albumin and IgG in CSF and serum were detected using Beckman Coulter IMMAGE800 automatic specific protein analyzer (United States, IMM-10013) and its original supporting reagents, and using the industry gold standard rate scattering method with high efficiency and accuracy. The reference ranges were all based on the biological reference intervals provided by BECKMAN COULTER and executed in accordance with the “Clinical Laboratory Testing Project Reference Interval Establishment Standard” formulated by the National Health Commission of the People’s Republic of China (WCH-LM-IMM-EXT-SP-026-07 WS/T 402). The reference ranges of CSF microprotein, albumin, IgG, index of IgG (IgG_index_), and serum IgG (s-IgG) were 0.15–0.45 g/L, 0.134–0.337 g/L, 0.005–0.041 g/L, 0.18–0.84, and 8.00–15.50 g/L. Q_ALB_ (Q_ALB_ = Alb_CSF_/Alb_S_) is the albumin quotient, recognized as an effective marker to evaluate the permeability of the blood–brain barrier (BBB) (Reiber and Peter, 2001). CSF IgG_index_ is calculated through the equation; IgG_index_ = Q_IgG_/Q_ALB_(Q_IgG_ = CSF IgG/s-IgG).

#### Statistics

The normal distribution of the data was assessed by visual inspection and Shapiro–Wilk tests. The levels of CSF profiles between ALS patients and control subjects were compared using the Mann–Whitney U test and Kruskal-Wallis nonparametric test for non-normal variables and the Student’s test for normal variables. The chi-square test was used to evaluate differences in frequencies. Pearson’s or Spearman’s correlation coefficients were used to evaluate the associations between these parameters and clinical phenotypes. One-way analysis of variance was used for comparison among multiple groups, and LSD and Welch’s ANOVA tests were used for pair-to-group comparison. Multiple regression analysis was applied to find these associations with adjustment for confounding factors. Survival analysis was performed with a stepwise Cox proportional hazard analysis. After propensity score matching of ALS and control participants based on age and sex in a 1:1 ratio, inter-group comparisons of CSF profiles were conducted once more. The results of non-normal data were presented as non-normal data as median (interquartile range). We utilized the Hodges–Lehmann estimation to compute the median difference along with the 95% CI. The significance level was set at *p* < 0.05. All statistical analyses were conducted with SPSS (v26; IBM SPSS Statistics for Windows, Armonk, NY) software and GraphPad Prism 9 (GraphPad Software, Inc. Boston, MA, United States).

## Results

In the study, 957 ALS patients underwent CSF and serum examinations during hospitalization. Eighty-seven ALS patients were lost to follow-up and were excluded from the study (9.1%) (Supplementary Figure S1). The control group consisted of 96 patients diagnosed with primary headache (41.7%) and anxiety and depression status (58.3%). The median age of ALS patients was 57.26 (48.15–64.99) years, while that of the control group was 41.00 (26.00–52.00) years. The ALS group comprised 530 males and 340 females, whereas the control group consisted of 28 males and 68 females.

Among the included 870 ALS patients, 69.1% were classical phenotype, 77.0% were of limb onset, 23.0% were of bulbar onset, 26.2% were at stage I, 39.8% were at stage II, and 34.0% were at stage III. As of August 2023, 43.1% of the patients had taken riluzole for more than 3 months, and 70.3% of the patients had died (with a median survival time of 42.43 months). The characteristics of male ALS and female ALS patients are listed in Supplementary Table S1, and male ALS patients were found to be older and had more limb onset, higher mortality rate, and lower median survival time than female ALS patients (Supplementary Table S1).

We compared the CSF profiles between the 870 ALS patients and 96 control subjects, and found that CSF microprotein, albumin, IgG, and Q_ALB_ in ALS patients were higher than those in the control group, but CSF IgG_index_ was lower than that in the control group (Table 1), although the difference was small. In ALS patients, the proportions above the median of the CSF microprotein, IgG, albumin, and Q_ALB_ were significantly higher than those in controls (CSF microprotein: 56.9% vs. 13.5%; IgG: 53.6% vs. 26.0%; albumin: 53.5% vs. 19.8%; Q_ALB_: 53.7% vs. 11.5%). In ALS patients, the proportion of the CSF microprotein exceeding the upper limit of the normal range was 32.6%, IgG was 26.1%, and albumin was 14.6%. Besides, we found that the difference in CSF microprotein, IgG, albumin, and Q_ALB_ remained significantly higher between ALS and controls in both male and female subgroups (Supplementary Tables S2, S3). However, the differences in the CSF IgG_index_ between female ALS and female control subjects were insignificant (Supplementary Table S3). Taking into account the significant differences in age and sex between ALS and control participants, propensity score matching was conducted based on age and sex, resulting in the selection of 69 ALS participants and 69 control participants in a 1:1 matching ratio. After matching, there were no significant differences in age and sex between the groups. However, the levels of CSF microprotein, IgG, albumin, and Q_ALB_ in the ALS group remained significantly higher than those in the control group, and the CSF IgG_index_ remained significantly lower than that in the control group, which were consistent with the results of the unmatched group comparisons (Table 1). In addition, in the subgroup analyses, we found that CSF microprotein, albumin, IgG, and Q_ALB_ were higher in male than in female participants (Supplementary Tables S4, S5).

**Table 1:** Comparison of CSF profiles between ALS and control groups.

	Non-matched				Matched		
	ALS (*N* = 870)	Controls (*N* = 96)	Estimated median difference (95% CI)	*p*	ALS (*N* = 69)	Controls (*N* = 69)	*p*
Age	57.26 (48.15–64.99)	41.00 (26.00–52.00)		**<0.001**	46.64 (39.10–62.44)	49.00 (37.50–57.00)	0.907
Sex (female%)	340 (39.1)	68 (70.8)		**<0.001**	32 (61.5)	46 (66.7)	0.378
CSF microprotein (g/L)	0.390 (0.330–0.470)**↑**	0.300 (0.243–0.340)	−0.100 (−0.120, −0.080)	**<0.001**	0.36 (0.30–0.45) ↑	0.32 (0.25–0.36)	**<0.001**
Proportion of CSF microprotein exceeding median (0.380 g/L)	56.9%**↑**	13.5%		**<0.001**	44.9%↑	15.9%	**<0.001**
Proportion of CSF microprotein exceeding the upper limit (0.450 g/L)	32.6%	0.0%			24.6%	0.0%	
CSF IgG (g/L)	0.030 (0.023–0.042)**↑**	0.022 (0.017–0.030)	−0.008 (−0.011, −0.006)	**<0.001**	0.03 (0.02–0.04) ↑	0.02 (0.02–0.03)	**0.002**
Proportion of CSF IgG exceeding median (0.029 g/L)	53.6%**↑**	26.0%		**<0.001**	53.6%**↑**	29.0%	**0.003**
Proportion of CSF IgG exceeding the upper limit (0.041 g/L)	26.1%	0.0%			27.5%	0.0%	
CSF albumin (g/L)	0.214 (0.174–0.278)**↑**	0.160 (0.128–0.200)	−0.057 (−0.071, −0.044)	**<0.001**	0.15 (0.20–0.26) ↑	0.14 (0.17–0.20)	**<0.001**
Proportion of CSF albumin exceeding median (0.207 g/L)	53.5%**↑**	19.8%		**<0.001**	47.8% ↑	23.2%	**0.002**
Proportion of CSF albumin exceeding the upper limit (0.337 g/L)	14.6%	0.0%			11.6%	0.0%	
CSF IgG_index_	0.494 (0.455–0.531) ↓	0.508 (0.467–0.543)	0.014 (0.000, 0.028)	**0.047**	0.50 (0.44–0.53) ↓	0.50 (0.45–0.56)	**0.029**
Proportion of CSF IgG_index_ exceeding median (0.495)	49.8%	55.2%		0.312	53.6%	50.7%	0.733
Proportion of CSF IgG_index_ exceeding the upper limit (0.840)	0.5%	0.0%			0.0%	0.0%	
Q_ALB_	0.006 (0.005–0.007)**↑**	0.004 (0.003–0.005)	−1.659 (−2.009, −1.311)	**<0.001**	0.005 (0.004–0.007)**↑**	0.004 (0.003–0.005)	**<0.001**
Proportion of CSF Q_ALB_ exceeding median (0.005)	53.7%**↑**	11.5%		**<0.001**	52.2%**↑**	15.9%	**<0.001**
s-IgG (g/L)	11.200 (9.625–12.700)	10.950 (9.530–12.450)	−0.300 (−0.800, 0.220)	0.304	11.25 (9.64–13.63)	10.80 (9.50–12.10)	0.060
Proportion of s-IgG exceeding median (11.100 g/L)	52.3%	49.0%		0.576	53.9%	46.4%	0.516
Proportion of s-IgG exceeding the upper limit (15.500 g/L)	3.1%	0.0%			7.7%	0.0%	

Bold: *p* < 0.05. Q_ALB_ = Alb_CSF_/Alb_S_ (CSF albumin/serum albumin); s-IgG: serum IgG; **↑:** CSF profiles in ALS group were higher than that in control group; ↓: CSF profiles in ALS group were lower than that in control group.

The correlation analyses found that CSF IgG was correlated with survival status (*r* = −0.075, *p* = 0.028; Table 2), that is, the higher the CSF IgG, the higher the risk of death from ALS. CSF IgG_index_ was negatively correlated with ALSFRS-R (*r* = −0.067, *p* = 0.048; Table 2). CSF IgG, albumin, and Q_ALB_ were correlated with disease duration (CSF IgG: *r* = −0.105, *p* = 0.002; CSF albumin: *r* = −0.146, *p* < 0.001; Q_ALB_: *r* = 0.155 *p* < 0.001; Table 2). Considering the influence of sex in CSF profiles, we stratified ALS patients according to sex and found that in male ALS patients, CSF IgG and albumin were correlated with survival status, and CSF IgG_index_ was negatively correlated with ALSFRS-R (*r* = −0.109, *p* = 0.012, Table 2), but these associations did not exist in female ALS. Besides, we found that the associations of CSF IgG, albumin, Q_ALB,_ and disease duration were found only in female ALS, but not in male ALS (Table 2).

**Table 2:** Correlation analysis between CSF profiles and clinical features of ALS.

*r*/*P*	Age	Sex	Disease stage	Site of onset	Disease duration	ALSFRS-R	Progression rate	Survival state
CSF microprotein	0.035/0.303	**−0.109/0.001**	**−0.071/0.036**	0.035/0.302	−0.021/0.535	0.010/0.777	0.012/0.728	−0.014/0.676
CSF IgG	−0.036/0.288	**−0.073/0.032**	−0.053/0.120	−0.032/0.348	**−0.105/0.002**	−0.011/0.738	−0.031/0.354	**−0.075/0.028**
CSF albumin	0.057/0.095	**−0.118/0.000**	−0.051/0.136	0.000/0.990	**−0.146/0.000**	0.015/0.655	−0.022/0.518	−0.040/0.240
CSF IgG_index_	0.020/0.549	−0.036/0.286	0.044/0.197	−0.020/0.548	0.014/0.690	**−0.067/0.048**	−0.007/0.839	0.034/0.313
Q_ALB_	**0.067/0.047**	**−0.109/0.001**	−0.047/0.164	0.000/0.992	**0.155/0.000**	0.013/0.712	−0.018/0.594	−0.001/0.979
**Male ALS**								
CSF microprotein	0.048/0.275		−0.053/0.223	0.019/0.657	0.007/0.886	−0.058/0.179	−0.002/0.972	−0.080/0.065
CSF IgG	0.037/0.396		−0.050/0.247	−0.054/0.218	−0.006/0.895	−0.067/0.126	−0.044/0.314	**−0.122/0.005**
CSF albumin	0.029/0.510		−0.017/0.697	0.007/0.879	0.004/0.926	−0.050/0.251	−0.009/0.828	**−0.113/0.009**
CSF IgG_index_	−0.003/0.939		0.074/0.090	−0.013/0.773	−0.035/0.426	**−0.109/0.012**	−0.005/0.910	0.028/0.520
Q_ALB_	0.054/0.213		−0.023/0.600	0.006/0.896	0.013/0.765	−0.049/0.264	−0.008/0.860	−0.072/0.097
**Female ALS**								
CSF microprotein	0.008/0.890		−0.068/0.212	0.031/0.565	−0.050/0.362	0.077/0.158	0.022/0.687	0.056/0.303
CSF IgG	0.032/0.557		−0.047/0.392	−0.021/0.705	**0.224/0.000**	0.044/0.419	−0.020/0.710	−0.024/0.655
CSF albumin	0.079/0.144		−0.082/0.133	−0.041/0.452	**0.246/0.000**	0.052/0.339	−0.033/0.547	0.042/0.442
CSF IgG_index_	−0.057/0.297		0.002/0.996	−0.040/0.458	0.093/0.086	−0.005/0.923	−0.013/0.816	0.034/0.531
Q_ALB_	0.085/0.116		−0.067/0.219	−0.036/0.506	**0.251/0.000**	0.044/0.423	−0.027/0.622	0.087/0.111

Bold: *p* < 0.05.

**Figure 1:**
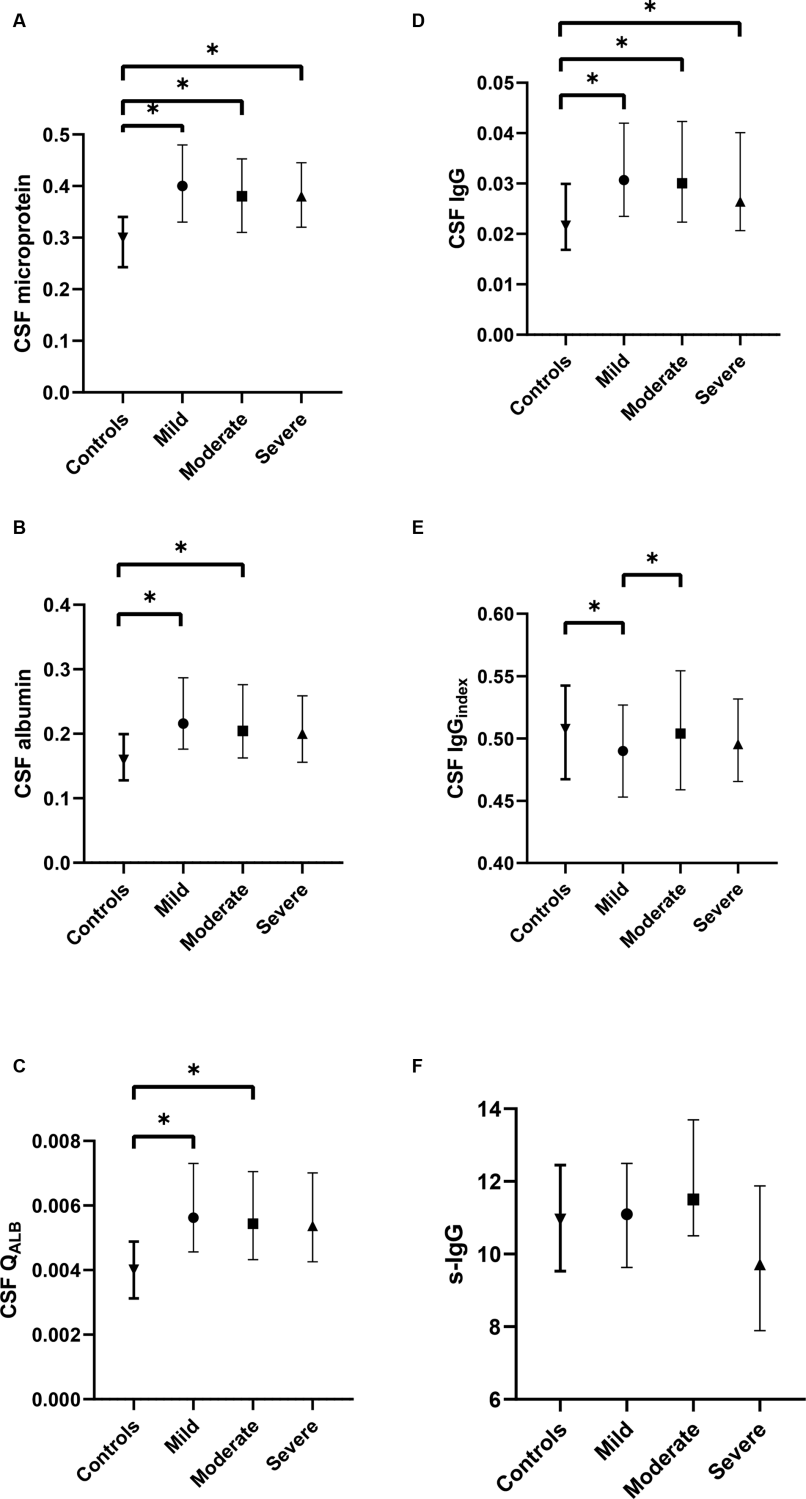
Comparison of CSF profiles among different subgroups of ALS and control group. **(A)** The difference in CSF microprotein, albumin, and IgG between mild, moderate, and severe ALS; **(B)** The difference in CSF albumin between mild, moderate, and severe ALS; **(C)** The difference in Q_ALB_ between mild, moderate, and severe ALS; **(D)** The difference in CSF IgG between mild, moderate, and severe ALS; **(E)** The difference in CSF IgG_index_ between mild, moderate, and severe ALS; **(F)** The difference in serum IgG between mild, moderate, and severe ALS. *p < 0.05.

In addition, we classified ALS into tertiles based on ALSFRS-R scores to analyze the correlation between CSF IgG_index_ and disease severity. We found that CSF IgG_index_ had significant differences between tertiles. Moderate ALS patients had higher CSF IgG_index_ compared to mild ALS patients (0.504 vs. 0.490, *p* = 0.006; Figure 1).

In order to verify whether the relationship between CSF IgG_index_ and ALSFRS-R was affected by other factors, multiple regression analysis was applied, and the results showed that CSF IgG_index_ was weakly correlated with ALSFRS-R scores in ALS patients after adjusting age of onset, sex, BMI, disease stage, site of onset, disease duration, and riluzole use (*F* = 30.624, *p* < 0.001; CSF IgG_index_: *β* = −0.062, *p* = 0.041; Table 3). In the subgroup analyses, these correlations still existed in male ALS patients (*β* = −0.095, *p* = 0.015; Supplementary Table S6), but not in female ALS patients (*β* = −0.014, *p* = 0.784; Supplementary Table S7).

**Table 3:** Multiple linear regression analysis of ALSFRS-R of CSF IgG_Index_.

	Unnormalized coefficient		Normalized coefficient	*t*	*p*	95.0% Confidence interval	
	Beta	Standard error	Beta			Lower limit	Upper limit
Age of onset	−0.070	0.019	−0.114	−3.685	**<0.001**	−0.107	−0.033
Sex	−0.764	0.427	−0.055	−1.792	0.073	−1.602	0.073
BMI	−0.039	0.070	−0.017	−0.552	0.581	−0.176	0.099
Disease stage	−3.707	0.270	−0.424	−13.754	**<0.001**	−4.237	−3.178
Site of onset	−0.138	0.187	−0.023	−0.739	0.460	−0.505	0.229
Disease duration	−0.062	0.014	−0.133	−4.281	**<0.001**	−0.090	−0.033
Riluzole use	1.768	0.417	0.130	4.242	**<0.001**	0.950	2.586
CSF IgG_index_	−5.091	2.481	−0.062	−2.052	**0.041**	−9.962	−0.220

Bold: *p* < 0.05.

**Figure 2:**
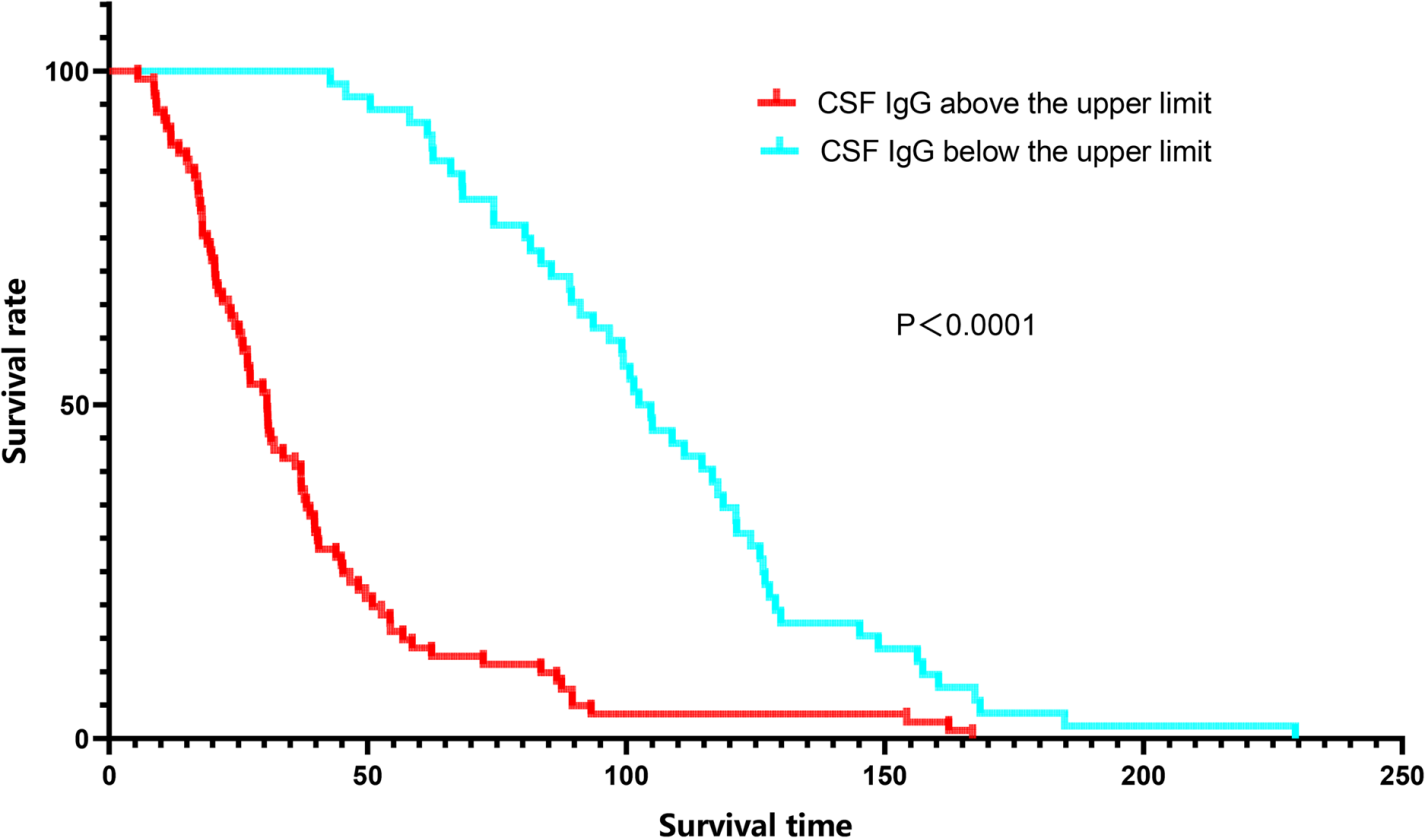
Kaplan–Meier survival curves of ALS patients according to CSF IgG stratifications. Red line: CSF IgG above the upper limit. Blue line: CSF IgG below the lower limit.

**Figure 3:**
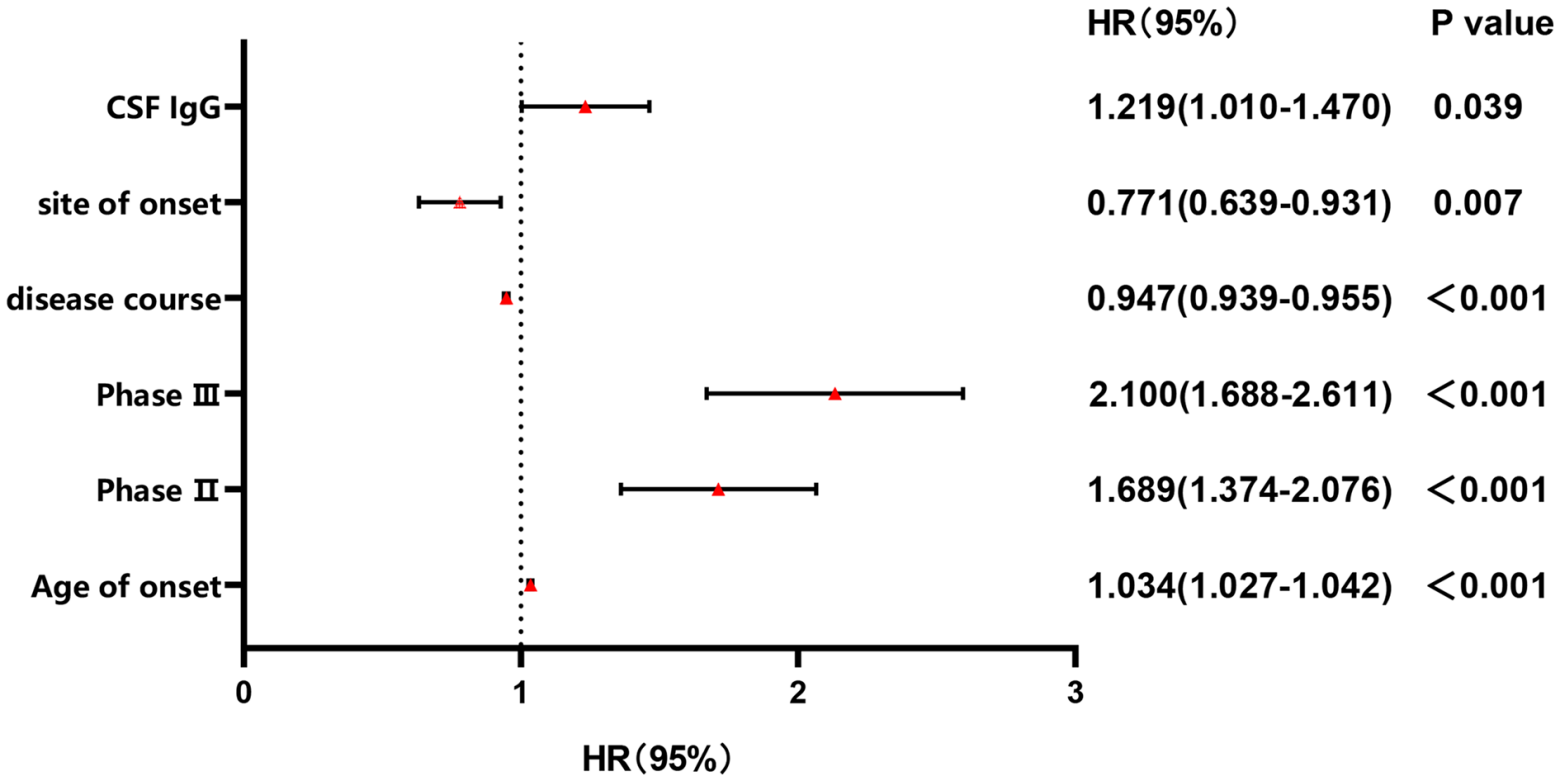
Forest plot of multivariable COX regression analysis for CSF IgG. Stage II: the odds ratio of mortality risk in Stage II compared to Stage I; Stage III: the odds ratio of mortality risk in Stage III compared to Stage I.

Since a significant correlation between CSF IgG and survival status was identified in ALS patients, we conducted further subgroup analysis based on the normal range of CSF IgG, and found that the survival time was shorter and survival rate were lower in the subgroup with CSF IgG exceeding the upper limit of the normal range (*p* < 0.001; Figure 2). In order to adjust the effects of other factors, including age of onset, disease course, disease stage, and site of onset, Cox survival analyses were performed. CSF IgG exceeding the upper limit of the normal range was significantly associated with the increased mortality risk in ALS patients [HR = 1.219 (1.010–1.470), *p* = 0.039; Figure 3]. Besides, ALS patients in stages II and III might be associated with increased mortality risk compared to those in stage I [stage II: HR = 1.689 (1.374–2.076), *p* < 0.001; stage III: HR = 2.100 (1.688–2.611), *p* < 0.001; Figure 3]. Older age of onset might be associated with increased mortality risk [age of onset: *HR* = 1.034 (1.027–1.042), *p* < 0.001; Figure 3], while longer disease duration and limb-onset compared to other onset regions may be associated with lower mortality risk [disease duration: HR = 0.947 (0.939–0.955), *p* < 0.001; site of onset: HR = 0.771 (0.639–0.931), *p* = 0.007; Figure 3].

Sensitivity analysis: First, we compared the levels of CSF profiles between the lost to follow-up ALS patients and ALS patients included in the analysis and found no significant differences between ALS patients who were lost to follow-up and ALS patients who completed follow-up (Supplementary Table S8). Secondly, we included the loss to follow-up ALS patients in the sensitivity analysis and found that it did not affect the results of the aforementioned analysis.

## Discussion

The study found a significant association between CSF IgG and survival status in ALS patients. The survival time was shorter and survival rates were lower in the ALS with CSF IgG exceeding the upper limit of the normal range. CSF IgG_index_ may negatively correlate with ALSFRS-R scores, especially in male ALS.

The current study found approximately one-third of ALS patients had higher CSF IgG levels. A previous study found IgG isolated from ALS patients increased the mobility of primary astrocyte endosomes and lysosomes, suggesting that it may be involved in the endocytosis/autophagy pathway (Stenovec et al., 2011). A single intraperitoneal injection of serum IgG from ALS patients induced a selective increase in the excitatory amino acids aspartate and glutamate levels in the CSF of rats (La Bella et al., 1997). Furthermore, some studies showed that IgG isolated from ALS patients can bind to CD16 receptors on microglia or lymphocytes and immune synapses between microglia and neurons (Edri-Brami et al., 2012, 2015), and can activate the oxidative stress response of microglia and release inflammatory factors (Milošević et al., 2017). Therefore, CSF IgG in ALS patients may accelerate the mortality risk in ALS by activating microglia to release oxidative stress. However, future basic research is still needed to elucidate the role of increased CSF IgG.

IgG_index_ = Q_IgG_/Q_ALB_(Q_IgG_ = CSF IgG/s-IgG, Q_ALB_ = Alb_CSF_/Alb_S_). In our study, we found that in mild ALS, the CSF IgG_index_ was lower compared to the control group. We considered this may be due to the significant increase in Q_ALB_ in the ALS group compared to the control group. Therefore, as Q_ALB_ serves as the divisor in the formula (IgG_index_ = Q_IgG_/Q_ALB_), its significant increase may lead to a decrease in the quotient. However, the difference in Q_ALB_ between mild ALS and moderate ALS was not significant. In this situation, CSF IgG_index_ may better reflect changes in Q_IgG_, as consistent with the finding of the negative association between CSF IgG_index_ and ALSFRS-R scores in ALS patients. That is, the more severe the ALS patient, the higher the CSF IgG_index_ may be, indicating potentially more intrathecal IgG synthesis. Therefore, as an accessible CSF biomarker, CSF IgG_index_ can only roughly reflect the situation of intrathecal synthesis, and compared to the most reliable indicator of intrathecal oligoclonal protein synthesis, OB, it definitely has its limitations.

Q_ALB_ is recognized as an effective marker to evaluate the permeability of the BBB (Reiber and Peter, 2001). The current finding of the significant increase in Q_ALB_ of ALS patients was consistent with some studies (Wu et al., 2020; Alarcan et al., 2023). Some researchers believed that the disruption of BBB was an early event in ALS, and most studies suggested that BBB disruption in neurodegenerative diseases mediates the invasion of immune cells from the blood, resulting in neurodegeneration (Aragón-González et al., 2022; Beaman et al., 2022). However, Sweeney et al. suggested that decreased CSF reabsorption and/or production may elevate Q_ALB_, leading to potential false-positive results in reflecting BBB breakdown (Sweeney et al., 2018). Therefore, further research is necessary to investigate the generation and absorption of CSF in ALS patients, as well as the extent of BBB disruption, for a comprehensive analysis of the role of the immune system in the occurrence and progression of ALS.

In the study, we found that CSF microprotein, IgG, albumin, and Q_ALB_ in male ALS patients were higher than those in female ALS patients, and the association between IgG_index_ and ALSFRS-R was found in male ALS patients, not in females. The male-to-female ratio of sporadic ALS is 1.3–1.5 (Logroscino et al., 2010). Hormones may play a protective role in the lower prevalence of ALS in females. The protective effect of female steroids may be due to their ability to prevent cell death and reduce the inflammatory component of the disease by acting directly on receptors expressed by motor neurons and muscle cells (Zhang et al., 2005). Therefore, sex differences should be paid attention to in the future.

## Limitation

There are some limitations in the current study. First of all, although the research findings of this study were significant, the associations were relatively diverse, and this study only represents the situation of the local cohort; therefore, further validation is required through longitudinal multi-center studies. Secondly, it was not combined with other inflammatory and immune-related biomarkers in blood and CSF to analyze its correlation and its role in the disease progression and prognosis of ALS. Third, the study was observational only, and studies on the mechanism of changes of CSF IgG involved in the pathophysiology of ALS are needed in the future.

## Conclusion

In the current study, we found that the higher CFS IgG was associated with increased mortality risk of ALS. Additionally, CSF IgG_index_ may be associated with the severity of ALS, especially in male ALS.

## Data availability statement

The original contributions presented in the study are included in the article/Supplementary material, further inquiries can be directed to the corresponding authors.

## Ethics statement

The studies involving humans were approved by the ethical standards committee on human experimentation at West China Hospital. The studies were conducted in accordance with the local legislation and institutional requirements. The participants provided their written informed consent to participate in this study.

## Author contributions

JF: Conceptualization, Data curation, Formal analysis, Investigation, Methodology, Software, Writing – original draft. XL: Conceptualization, Data curation, Formal analysis, Investigation, Software, Writing – original draft. QW: Conceptualization, Data curation, Investigation, Writing – original draft. XC: Conceptualization, Data curation, Investigation, Writing – original draft. HS: Conceptualization, Funding acquisition, Supervision, Writing – review & editing.

## References

[ref1] Akçimen F. Lopez E. R. Landers J. E. Nath A. Chiò A. Chia R.. (2023). Amyotrophic lateral sclerosis: translating genetic discoveries into therapies [published online ahead of print, 2023 Apr 6]. Nat. Rev. Genet. 24, 642–658. doi: 10.1038/s41576-023-00592-y, PMID: 37024676 PMC10611979

[ref2] Alarcan H. Vourc’h P. Berton L. Benz-de Bretagne I. Piver E. Andres C. R.. (2023). Implication of central nervous system barrier impairment in amyotrophic lateral sclerosis: gender-related difference in patients. Int. J. Mol. Sci. 24:11196. doi: 10.3390/ijms241311196, PMID: 37446372 PMC10342931

[ref3] Aragón-González A. Shaw P. J. Ferraiuolo L. (2022). Blood-brain barrier disruption and its involvement in neurodevelopmental and neurodegenerative disorders. Int. J. Mol. Sci. 23:15271. doi: 10.3390/ijms232315271 36499600 PMC9737531

[ref4] Assialioui A. Domínguez R. Ferrer I. Andrés-Benito P. Povedano M. (2022). Elevated cerebrospinal fluid proteins and albumin determine a poor prognosis for spinal amyotrophic lateral sclerosis. Int. J. Mol. Sci. 23:11063. doi: 10.3390/ijms231911063, PMID: 36232365 PMC9570498

[ref5] Beaman C. Kozii K. Hilal S. Liu M. Spagnolo-Allende A. J. Polanco-Serra G.. (2022). Cerebral microbleeds, cerebral amyloid Angiopathy, and their relationships to quantitative markers of neurodegeneration. Neurology 98, e1605–e1616. doi: 10.1212/WNL.0000000000200142, PMID: 35228332 PMC9052569

[ref6] Chen L. Xu L. Tang L. Xia K. Tian D. Zhang G.. (2021). Trends in the clinical features of amyotrophic lateral sclerosis: a 14-year Chinese cohort study. Eur. J. Neurol. 28, 2893–2900. doi: 10.1111/ene.14943, PMID: 34048130

[ref7] Edri-Brami M. Rosental B. Hayoun D. Welt M. Rosen H. Wirguin I.. (2012). Glycans in sera of amyotrophic lateral sclerosis patients and their role in killing neuronal cells. PLoS One 7:e35772. doi: 10.1371/journal.pone.0035772, PMID: 22666317 PMC3364259

[ref8] Edri-Brami M. Sharoni H. Hayoun D. Skutelsky L. Nemirovsky A. Porgador A.. (2015). Development of stage-dependent glycans on the fc domains of IgG antibodies of ALS animals. Exp. Neurol. 267, 95–106. doi: 10.1016/j.expneurol.2015.02.023, PMID: 25725350

[ref9] Gårde A. Kjellin K. G. (1971). Diagnostic significance of cereborspinal-fluid examinations in myelopathy. Acta Neurol. Scand. 47, 555–568. doi: 10.1111/j.1600-0404.1971.tb07508.x 4110749

[ref10] Guiloff J. F. McGregor B. Thompson E. Blackwood W. Paul E. (1979). Age and cerebrospinal-fluid protein in motor-neuron disease. N. Engl. J. Med. 300, 437–438. doi: 10.1056/nejm197902223000820 759927

[ref11] Guiloff R. J. McGregor B. Thompson E. Blackwood W. Paul E. (1980). Motor neurone disease with elevated cerebrospinal fluid protein. J. Neurol. Neurosurg. Psychiatry 43, 390–396. doi: 10.1136/jnnp.43.5.390, PMID: 7420088 PMC490564

[ref12] Klose V. Jesse S. Lewerenz J. Kassubek J. Dorst J. Tumani H.. (2023). CSF oligoclonal IgG bands are not associated with ALS progression and prognosis. Front. Neurol. 14:1170360. Published 2023 May 5. doi: 10.3389/fneur.2023.1170360, PMID: 37213901 PMC10196068

[ref13] La Bella V. Goodman J. C. Appel S. H. (1997). Increased CSF glutamate following injection of ALS immunoglobulins. Neurology 48, 1270–1272. doi: 10.1212/wnl.48.5.1270, PMID: 9153455

[ref14] Leonardi A. Abbruzzese G. Arata L. Cocito L. Vische M. (1984). Cerebrospinal fluid (CSF) findings in amyotrophic lateral sclerosis. J. Neurol. 231, 75–78. doi: 10.1007/BF00313720 6737012

[ref15] Li J. Y. Cai Z. Y. Sun X. H. Shen D. C. Yang X. Z. Liu M. S.. (2022). Blood-brain barrier dysfunction and myelin basic protein in survival of amyotrophic lateral sclerosis with or without frontotemporal dementia. Neurol. Sci. 43, 3201–3210. doi: 10.1007/s10072-021-05731-z 34826032

[ref16] Li C. Liu J. Lin J. Shang H. (2022). COVID-19 and risk of neurodegenerative disorders: a Mendelian randomization study. Transl. Psychiatry 12:283. doi: 10.1038/s41398-022-02052-3 35835752 PMC9281279

[ref17] Logroscino G. Traynor B. J. Hardiman O. Chio A. Mitchell D. Swingler R. J.. (2010). Incidence of amyotrophic lateral sclerosis in Europe. J. Neurol. Neurosurg. Psychiatry 81, 385–390. doi: 10.1136/jnnp.2009.183525, PMID: 19710046 PMC2850819

[ref18] Milošević M. Milićević K. Božić I. Lavrnja I. Stevanović I. Bijelić D.. (2017). Immunoglobulins G from sera of amyotrophic lateral sclerosis patients induce oxidative stress and upregulation of Antioxidative system in BV-2 microglial cell line. Front. Immunol. 8:1619. Published 2017 Nov 23. doi: 10.3389/fimmu.2017.01619, PMID: 29218049 PMC5703705

[ref19] Norris F. H. Burns W. U K. S. Mukai E. Norris H. (1993). Spinal fluid cells and protein in amyotrophic lateral sclerosis. Arch. Neurol. 50, 489–491. doi: 10.1001/archneur.1993.00540050041012 8489404

[ref20] Reiber H. Peter J. B. (2001). Cerebrospinal fluid analysis: disease-related data patterns and evaluation programs. J. Neurol. Sci. 184, 101–122. doi: 10.1016/s0022-510x(00)00501-3, PMID: 11239944

[ref21] Rosenbohm A. Liu M. Nagel G. Peter R. S. Cui B. Li X.. (2018). Phenotypic differences of amyotrophic lateral sclerosis (ALS) in China and Germany. J. Neurol. 265, 774–782. doi: 10.1007/s00415-018-8735-9 29392461

[ref22] Stenovec M. Milošević M. Petrušić V. Potokar M. Stević Z. Prebil M.. (2011). Amyotrophic lateral sclerosis immunoglobulins G enhance the mobility of Lysotracker-labelled vesicles in cultured rat astrocytes. Acta Physiol (Oxf.) 203, 457–471. doi: 10.1111/j.1748-1716.2011.02337.x, PMID: 21726417

[ref23] Sweeney M. D. Sagare A. P. Zlokovic B. V. (2018). Blood-brain barrier breakdown in Alzheimer disease and other neurodegenerative disorders. Nat. Rev. Neurol. 14, 133–150. doi: 10.1038/nrneurol.2017.188, PMID: 29377008 PMC5829048

[ref24] Tarasiuk J. Kułakowska A. Drozdowski W. Kornhuber J. Lewczuk P. (2012). CSF markers in amyotrophic lateral sclerosis. J. Neural Transm. (Vienna) 119, 747–757. doi: 10.1007/s00702-012-0806-y 22555610

[ref25] Wei Q. Q. Hou Y. B. Zhang L. Y. Ou R. W. Cao B. Chen Y. P.. (2022). Neutrophil-to-lymphocyte ratio in sporadic amyotrophic lateral sclerosis. Neural Regen. Res. 17, 875–880. doi: 10.4103/1673-5374.322476, PMID: 34472488 PMC8530123

[ref26] Wu Y. Yang X. Li X. Wang H. Wang T. (2020). Elevated cerebrospinal fluid homocysteine is associated with blood-brain barrier disruption in amyotrophic lateral sclerosis patients. Neurol. Sci. 41, 1865–1872. doi: 10.1007/s10072-020-04292-x, PMID: 32086685

[ref27] Yang T. Wei Q. Li C. Cao B. Ou R. Hou Y.. (2022). Spatial-temporal pattern of propagation in amyotrophic lateral sclerosis and effect on survival: a cohort study. Eur. J. Neurol. 29, 3177–3186. doi: 10.1111/ene.15527, PMID: 35996987

[ref28] Yang T. Xiao Y. Cheng Y. Huang J. Wei Q. Li C.. (2023). Epigenetic clocks in neurodegenerative diseases: a systematic review. J. Neurol. Neurosurg. Psychiatry 94, 1064–1070. doi: 10.1136/jnnp-2022-330931, PMID: 36963821

[ref29] Zhang R. Gascon R. Miller R. G. Gelinas D. F. Mass J. Hadlock K.. (2005). Evidence for systemic immune system alterations in sporadic amyotrophic lateral sclerosis (sALS). J. Neuroimmunol. 159, 215–224. doi: 10.1016/j.jneuroim.2004.10.009, PMID: 15652422

[ref30] Zhao X. Yang F. Wang H. Cui F. Li M. Sun B.. (2020). The increase in CSF total protein and immunoglobulins in Chinese patients with sporadic amyotrophic lateral sclerosis: a retrospective study. J. Neurol. Sci. 414:116840. doi: 10.1016/j.jns.2020.116840, PMID: 32388062

