# Supplementary material for: Associations of cerebrospinal fluid profiles with severity and mortality risk of amyotrophic lateral sclerosis
Source: Front Neurosci. 2024 May 15;18:1375892. doi: 10.3389/fnins.2024.1375892 (PMC11133581; doi:10.3389/fnins.2024.1375892)
Supplement: Supplementary file 1 [file Data_Sheet_1.docx]

Figure S1: Flow diagram


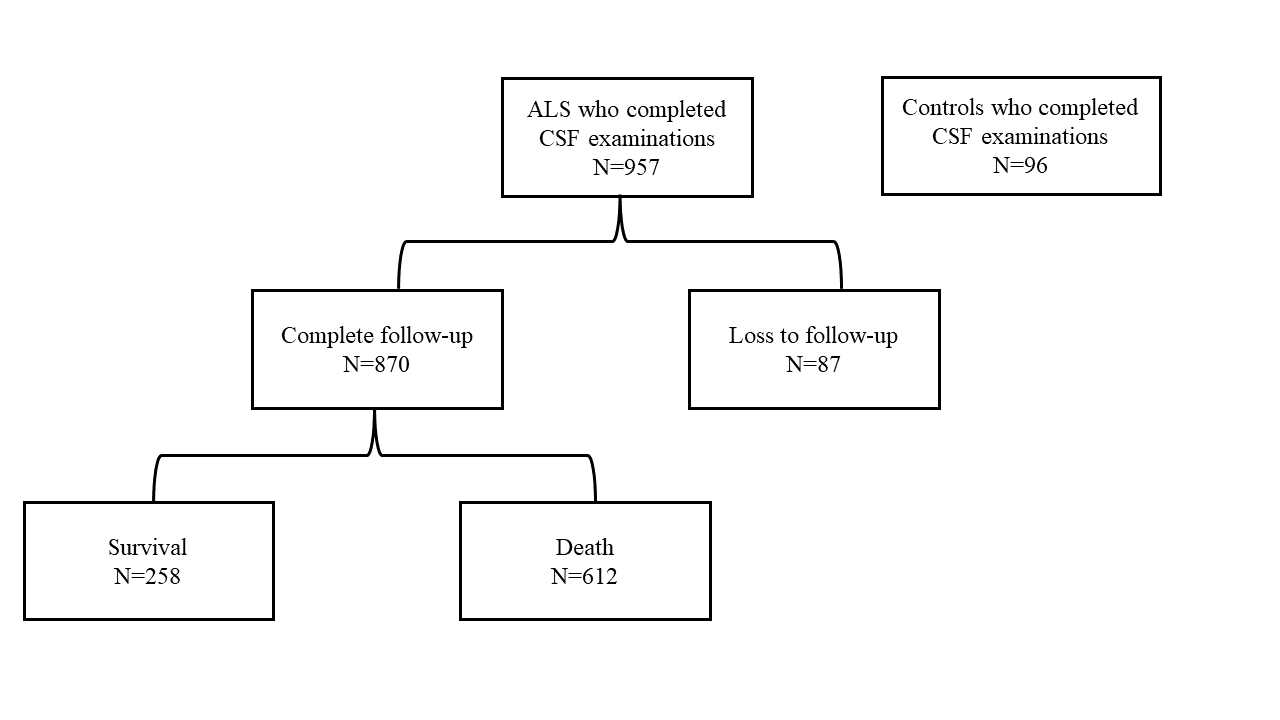


Table S1: Comparison of clinical features of male ALS and female ALS

|  | Male ALS(N=530) | Female ALS(N=340) | *P* |
| --- | --- | --- | --- |
| Age | 58.85(49.61-65.40) | 55.23(46.73-63.91) | **0.001** |
| Age of onset | 56.68(47.72-63.56) | 53.59(44.63-62.01) | **<0.001** |
| BMI | 22.00(20.24-24.03) | 21.54(20.06-24.22) | 0.231 |
| Classical phenotype (%) | 70.8% | 66.5% | 0.182 |
| Disease duration | 12.27(7.54-20.84) | 12.61(8.01-20.29) | 0.521 |
| Disease stage |  |  | 0.080 |
| stage Ⅰ (%) | 26.4% | 25.9% |  |
| stage Ⅱ (%) | 42.3% | 35.9% |  |
| stage Ⅲ (%) | 31.3% | 38.2% |  |
| Site of onset |  |  | **0.001** |
| bulbar onset (%) | 19.2% | 28.8% |  |
| Limb onset (%) | 80.8% | 71.2% |  |
| ALSFRS-R | 40.00(36.00-43.00) | 39.00(34.00-43.00) | 0.095 |
| progression rate | 1.15(0.68-1.97) | 1.10(0.61-1.82) | 0.086 |
| Survival state (death) (%) | 73.8% | 65.0% | **0.006** |
| median survival time (m) | 40.030 | 43.500 | **0.015** |

Notes: Bold: *P*<0.05.

Table S2: Comparison of CSF profiles between male ALS and male control subjects

|  | ALS (N=530) | Controls (N=28) | *P* |
| --- | --- | --- | --- |
| Age | 58.85(49.61-65.40) | 46.00(26.00-54.25) | **<0.001** |
| CSF microprotein (g/L) | 0.400(0.330-0.490)**↑** | 0.325(0.273-0.368) | **<0.001** |
| Proportion of CSF microprotein exceeding the upper limit (0.450 g/L) | 37.2% | 0.0% |  |
| CSF IgG (g/L) | 0.031(0.023-0.044)**↑** | 0.027(0.021-0.033) | **0.015** |
| Proportion of CSF IgG exceeding the upper limit (0.041g/L) | 29.1% | 0.0% |  |
| CSF albumin (g/L) | 0.222(0.177-0.299)**↑** | 0.193(0.150-0.238) | **0.005** |
| Proportion of CSF albumin exceeding the upper limit (0.337g/L) | 16.8%**↑** | 0.0% |  |
| CSF IgG_index_ | 0.496(0.457-0.534)↓ | 0.530(0.487-0.560) | **0.004** |
| Proportion of CSF IgG_index_ exceeding the upper limit (0.840) | 0.6% | 0.0% |  |
| Q_ALB_ | 0.006(0.005-0.008)**↑** | 0.005(0.004-0.005) | **<0.001** |
| s-IgG (g/L) | 11.050(9.473-12.100) | 10.350(8.658-12.900) | 0.463 |

Notes: Bold: *P*<0.05.

Table S3: Comparison of CSF profiles between female ALS and female control subjects

|  | ALS (N=340) | Controls (N=68) | *P* |
| --- | --- | --- | --- |
| Age | 55.23(46.73-63.91) | 40.00(26.00-52.00) | **<0.001** |
| CSF microprotein (g/L) | 0.380(0.320-0.450)**↑** | 0.280(0.220-0.338) | **<0.001** |
| Proportion of CSF microprotein exceeding the upper limit (0.450 g/L) | 25.6% | 0.0% |  |
| CSF IgG (g/L) | 0.029(0.022-0.039)**↑** | 0.020(0.016-0.028) | **<0.001** |
| Proportion of CSF IgG exceeding the upper limit (0.041g/L) | 21.5% | 0.0% |  |
| CSF albumin (g/L) | 0.203(0.166-0.260)**↑** | 0.147(0.119-0.181) | **<0.001** |
| Proportion of CSF albumin exceeding the upper limit (0.337g/L) | 11.2% | 0.0% |  |
| CSF IgG_index_ | 0.491(0.451-0.528) | 0.494(0.460-0.530) | 0.455 |
| Proportion of CSF IgG_index_ exceeding the upper limit (0.840) | 0.3% | 0.0% |  |
| Q_ALB_ | 0.005(0.004-0.007)**↑** | 0.004(0.003-0.005) | **<0.001** |
| s-IgG (g/L) | 11.300(10.070-13.100) | 11.300(9.575-12.450) | 0.156 |

Notes: Bold: *P*<0.05.

Table S4: Comparison of CSF profiles between male ALS and female ALS

|  | Male ALS(N=530) | Female ALS(N=340) | *P* |
| --- | --- | --- | --- |
| Age | 58.85(49.61-65.40) | 55.23(46.73-63.91) | **0.001** |
| CSF microprotein (g/L) | 0.400(0.330-0.490)**↑** | 0.380(0.320-0.450) | **0.001** |
| Proportion of CSF microprotein exceeding the upper limit (0.450 g/L) | 37.2%**↑** | 25.6% | **<0.001** |
| CSF IgG (g/L) | 0.031(0.023-0.044)**↑** | 0.029(0.022-0.039) | **0.032** |
| Proportion of CSF IgG exceeding the upper limit (0.041g/L) | 29.1%**↑** | 21.5% | **0.013** |
| CSF albumin (g/L) | 0.222(0.177-0.299)**↑** | 0.203(0.166-0.260) | **0.001** |
| Proportion of CSF albumin exceeding the upper limit (0.337g/L) | 16.8%**↑** | 11.2% | **0.022** |
| CSF IgG_index_ | 0.496(0.457-0.534) | 0.491(0.451-0.528) | 0.286 |
| Proportion of CSF IgG_index_ exceeding the upper limit (0.840) | 0.6% | 0.3% | 0.563 |
| Q_ALB_ | 0.006(0.005-0.008)**↑** | 0.005(0.004-0.007) | **0.001** |
| s-IgG (g/L) | 11.050(9.473-12.100) | 11.300(10.070-13.100) | 0.055 |

Notes: Bold: *P*<0.05. **↑:** CSF profiles in male ALS were higher than that in female ALS.

Table S5: Comparison of CSF profiles between male and female control subjects

|  | Male controls (N=28) | Female controls (N=68) | *P* |
| --- | --- | --- | --- |
| Age | 46.00(26.00-54.25) | 40.00(26.00-52.00) | 0.840 |
| CSF microprotein (g/L) | 0.325(0.273-0.368)**↑** | 0.280(0.220-0.338) | **0.021** |
| CSF IgG (g/L) | 0.027(0.021-0.033)**↑** | 0.020(0.016-0.028) | **0.004** |
| CSF albumin (g/L) | 0.193(0.150-0.238)**↑** | 0.147(0.119-0.181) | **0.001** |
| CSF IgG_index_ | 0.530(0.487-0.560)↓ | 0.494(0.460-0.530) | **0.021** |
| Q_ALB_ | 0.005(0.004-0.005)**↑** | 0.004(0.003-0.005) | **0.004** |
| s-IgG (g/L) | 10.350(8.658-12.900) | 11.300(9.575-12.450) | 0.486 |

Notes: Bold: *P*<0.05.

Table S6: Multiple linear regression analysis of ALSFRS-R in male ALS

|  | Unnormalized coefficient | | normalized coefficient | *t* | *P* | 95.0% Confidence interval | |
| --- | --- | --- | --- | --- | --- | --- | --- |
|  | Beta | standard error | Beta |  |  | Lower limit | Upper limit |
| Age of onset | -0.074 | 0.024 | -0.121 | -3.109 | **0.002** | -0.212 | -0.027 |
| BMI | -0.062 | 0.086 | -0.028 | -0.725 | **0.469** | -0.230 | 0.106 |
| Disease stage | -3.581 | 0.337 | -0.417 | -10.628 | **<0.001** | -4.243 | -2.919 |
| site of onset | -0.022 | 0.233 | 0.004 | 0.094 | 0.925 | -0.437 | 0.480 |
| Disease duration | -0.064 | 0.018 | -0.139 | -3.544 | **<0.001** | -0.100 | -0.029 |
| riluzole use | 1.786 | 0.514 | 0.135 | 3.476 | **0.001** | 0.776 | 2.919 |
| CSF IgG_index_ | -7.139 | 2.917 | -0.095 | -2.447 | **0.015** | -12.871 | -1.407 |

Notes: Bold: *P*<0.05.

Table S7: Multiple linear regression analysis of ALSFRS-R in female ALS

|  | Unnormalized coefficient | | normalized coefficient | *t* | *P* | 95.0% Confidence interval | |
| --- | --- | --- | --- | --- | --- | --- | --- |
|  | Beta | standard error | Beta |  |  | Lower limit | Upper limit |
| Age of onset | -0.063 | 0.032 | -0.101 | -2.004 | **0.046** | -0.125 | -0.001 |
| BMI | -0.003 | 0.121 | -0.001 | -0.021 | **0.983** | -0.240 | 0.235 |
| Disease stage | -3.858 | 0.451 | -0.432 | -8.552 | **<0.001** | -4.746 | -2.970 |
| site of onset | -0.349 | 0.316 | -0.056 | -1.106 | 0.270 | -0.970 | 0.272 |
| Disease duration | -0.060 | 0.024 | -0.129 | -2.506 | **0.013** | -0.108 | -0.013 |
| riluzole use | 1.673 | 0.719 | 0.117 | 2.326 | **0.021** | 0.257 | 3.089 |
| CSF IgG_index_ | -1.272 | 4.642 | -0.014 | -0.274 | 0.784 | -10.407 | 7.863 |

Notes: Bold: *P*<0.05.

Table S8: Comparison of CSF profiles between the lost to follow-up ALS patients and ALS patients included in the analysis

|  | the loss to follow-up ALS patients (N=870) | ALS patients included in the analysis (N=87) | *P* |
| --- | --- | --- | --- |
| CSF microprotein (g/L) | 0.390(0.330-0.470) | 0.410(0.320-0.520) | 0.457 |
| CSF IgG (g/L) | 0.030(0.023-0.042) | 0.029(0.022-0.044) | 0.888 |
| CSF albumin (g/L) | 0.214(0.174-0.278) | 0.217(0.164-0.302) | 0.875 |
| CSF IgG_index_ | 0.494(0.455-0.531) | 0.498(0.462-0.560) | 0.113 |
| Q_ALB_ | 0.006(0.005-0.007) | 0.006(0.004-0.008) | 0.977 |
| s-IgG (g/L) | 11.200(9.625-12.700) | 10.700(8.780-11.750) | 0.101 |

Notes: Bold: *P*<0.05.
